# Widefield microscopy for live imaging of lipid domains and membrane dynamics

**DOI:** 10.1016/j.bbamem.2010.11.017

**Published:** 2011-03

**Authors:** Guy Wheeler, Kevin M. Tyler

**Affiliations:** BioMedical Research Centre, Norwich School of Medicine, University of East Anglia, Norwich, NR4 7TJ, UK

**Keywords:** GP, generalized polarization, l_o_, liquid-ordered, MDCKII, Madin–Darby Canine Kidney II, Laurdan, Lipid raft, Widefield fluorescent imaging

## Abstract

Within the lateral organisation of plasma membranes of polarized cell types there exist heterogenous microdomains of distinct lipid composition, the small size of which (10–200 nm) makes them difficult to discern with traditional microscopic techniques, but which can be distinguished on the basis of lipid packing. These microdomains or rafts can be concentrated in larger more visible liquid-ordered regions, particularly by cross-linking of their constituents as in the immunological synapse or in features of the polarized cell such as pseudopodia or flagella. One technique, Laurdan fluorescence microscopy, has proven very useful for distinguishing such regions but has hitherto relied on 2-photon confocal microscopy. This has to some extent limited its utility to living systems and its widespread adoption in studying membrane dynamics on the surface of living cells. Here we describe and validate the adaptation of a standard widefield fluorescence microscope for live imaging of Laurdan stained cell membranes.

## Research Highlights

► Adaptation of wide-field microscope setup for visualising liquid-ordered domains. ► Real-time live cell imaging of plasma membrane heterogeneity and dynamics. ► Multi-channel simultaneous acquisition of biological markers with GP images.

## 1 Introduction

Cell membranes contain many different lipid species, with different combinations found in different cell types. Within cells there exists a heterogeneity of lipid species in the lateral organisation of the cell membrane, with distinct regions, or domains possessing different lipid composition. The possibility of lateral phase separation in phospholipids membranes has long been discussed [1], with Ipsen and Mouritsen [2] introducing the possibility of stable lipid domains existing within the cell membrane which exhibit liquid-ordered (l_o_) states relative to adjacent liquid-disordered regions (l_d_). These dynamic micro-domains are composed predominantly of cholesterol and glycosphingolipids and appear to mediate cellular processes such as signal transduction, protein sorting, cell adhesion, and membrane trafficking [3] and have been termed lipid rafts [4]. They were defined by Pike [5] as ‘small (10–200 nm), heterogeneous, highly dynamic, sterol- and sphingolipid-enriched domains that compartmentalise cellular processes.’ Components associated with lipid rafts have (controversially) been identified by their insolubility in detergents, and the association of these regions with the accumulation of raft-associated proteins [6]. The lipid raft hypothesis, although gaining increasing acceptance, is still the subject of some debate, in part due to the small size and transient nature of the rafts [6,7] and the difficulty in finding defined phase boundaries in living systems due to most biological membranes not being at equilibrium [8,9]. The size of the lipid raft, as defined by Pike [5] is also part of this debate, with the size definition depending on the technique being used to measure it. Their presence is often correlated to the clustering of many important signalling molecules, rather than by direct observation of the lipid organisation, due to the difficulty of demonstrating the assembly of distinct lipid species at these regions, with some postulating that the nanometre scale rafts are maintained by the cell as part of a mosaic of domains but are largely non-functional until they form larger, micrometre scale clusters around raft-associated proteins, as reviewed by Mayor and Rao, 2004 [10]. It therefore remains an important experimental goal to provide a correlation between the localised fluctuation of lipid composition and the cellular processes they are proposed to help mediate. However, it is clear that the segregation of cholesterol and sphingolipids is important in the compartmentalisation of membrane function [7]. Work on T-cell activation domains [11,12] has shown the functional necessity for these lipid species to cluster into domains [13] and the apparent role of lipid rafts in pathogen entry into cells [14,15], while recent reports have also revealed an association between membrane rafts and the haemagglutinin of influenza viruses [16]. Consequently, methods for studying these lipid rafts and their physiological roles have an increasing importance and are becoming increasingly studied. Jacobson et al. [17] and Lagerholm et al. [18] reviewed many of the techniques used for lipid raft and microdomain investigation.

The fluorescent probe 6-dodecanyl-2-dimethylaminonaphthalene (Laurdan) has been used to study higher-order lipid microdomains, due to its phase variation in ordered and fluid membrane regions. Laurdan is a lipophilic polarity-sensitive dye, first designed and synthesised by Weber and Farris [19], that incorporates into the membrane with an even distribution and is not influenced by cell surface modifications such as the binding of lipoproteins. Its dipole aligns parallel to the hydrophobic lipid chains of the membrane [20] and its usefulness for the study of membrane dynamics arises from the fact that its fluorescence changes depending on the amount of free water in the membrane. Laurdan reflects the water content of, and penetration into the surrounding membrane, rather than its specific lipid species composition [20]. In a membrane in a more fluid state, Laurdan fluoresces with greater intensity at green wavelengths, with an emission maximum centred at 490 nm, whereas in more ordered regions its emission maximum shifts to the blue wavelengths, centred at 440 nm. Generalised Polarisation (GP) ratiometric analysis was designed [21,22] to calculate the different lateral packing of membrane regions from the emission intensity at these two wavelengths, using the following equation:

$$GP=\left(I_{440}–I_{490}\right)/\left(I_{440}+I_{490}\right)$$

where I_440_ and I_490_ are the fluorescence intensities at those wavelengths. This results in a value for each pixel ranging from − 1.0 (most fluid) to + 1.0 (most ordered). Because the GP is a comparative ratio it is independent of local probe concentration, provided probe concentration is low enough to avoid quenching and any influence on the acyl chains in the membrane. The solution of probe and vehicle can also potentially influence the penetration of water into the membrane, altering the resulting GP.

Laurdan GP can be obtained using both spectroscopy and microscopy techniques [23] with the former a more general method for detecting trends in GP for whole samples such as the changes in spermatozoa under different conditions [24] and in the formation of endosomes [25] found in the trans-Golgi network. Spectrophotometers have also been used to study the effect of alcohol on erythrocyte membranes [26] where sample GP averages were reduced with increases in alcohol concentration. In microscopy, Laurdan staining has been used to study the ordered state of domains in artificial and physiological membranes. Giant unilamellar vesicles (GUVs) are a simplified system used to study phase separation of artificial lipid mixtures in response to temperature and pH changes [27–29] showing that at certain temperatures these artificial vesicles exhibit phase coexistence over their surface. For interactions with unlabelled proteins they can be used to observe whether the proteins cause any alterations in membrane phases [30]. Many of the proposed properties of lipid rafts have also been observed in these artificial membrane systems [31,32].

Kaiser et al. [33] used Laurdan microscopy to highlight differences in applicability between model and cell membranes, and the separation of different membrane proteins into regions of higher or lower order. In physiological systems, Laurdan microscopy has proved its usefulness in showing distinct clustering of Caveolin-1 with membrane domains of increased order [34] and has shown the increased order of the lamellopodia of macrophages [35] compared to other regions of the cell's membrane. Similarly, Gaus et al. [36] have demonstrated that focal adhesions are highly organised regions of the membrane.

The majority of previous reports have used two-photon microscopy, as this does not cause excessive photobleaching [37] to which Laurdan is particularly susceptible [38]. We report on the validation of a new widefield microscopy set-up with the ability to capture images in three different wavelengths simultaneously, and its potential use in the study of membrane dynamics in live cells in culture in response to the addition of various factors. We demonstrate its ability to capture Laurdan fluorescent images with probe concentrations close to those used for two-photon microscopy and the ability of the system to detect changes in membrane fluidity with minimal photobleaching. For this we show here our work on two cell lines of different physiological functions used in our laboratory, that have previously been shown to exhibit regions of higher and lower membrane order, and functional cell membrane protrusions such as macrophage filopodia. We also show the increased order associated with the protozoan flagellum. We also show its utility for using the third channel to monitor in the red wavelengths, using fluorescent beads coated with protein to show localised membrane order changes at the synapse, showing how our system can detect regions of different chain order values in response to extracellular stimuli.

## 2 Materials and methods

### 2.1 Cell culture

Madin–Darby Canine Kidney II (MDCKII) cells and murine macrophage-like RAW264.7 cells were grown in Dulbecco's Modified Eagle Medium (DMEM) with 10% foetal calf serum, at 37 °C and 5% CO_2_. Cells were plated out onto 4-well chamber coverslips (Nunc) at an initial density of 2–4 × 10^5^ cells mL^− 1^ for subsequent Laurdan staining.

*Trypanosoma rangeli* were grown in Liver Infused Tryptone (LIT) medium at 27.5 °C as previously described [43].

### 2.2 Laurdan staining

Membrane order was evaluated using the fluorescent probe 6-dodecanoyl-2-dimethylaminonapthalene (Laurdan) (Roche, UK). Laurdan was solubilised in dimethylsulfoxide (DMSO) to a stock concentration of 1 mM and kept at room temperature in the dark. This was kept for no longer than one month before being replaced with fresh stock solution.

For mammalian cells, culture medium was refreshed and Laurdan added to the required concentration in the dark to avoid photobleaching. For optimisation of Laurdan staining for mammalian cells final concentrations of 2, 5, 7.5, 10, 12.5, 15 and 20 μM Laurdan were used. Incubation times of 10, 15, 20, 30 and 45 min were trialled. After optimisation, cells were incubated in the dark with gentle rocking for fifteen minutes with the optimised Laurdan concentration to allow incorporation of the probe. The medium was then removed and the cells washed gently with 1× phosphate buffered saline solution (PBS) before a final volume of fresh medium was added. Chamber coverslips were then sealed and transferred to the microscope stage.

For *T. rangeli*, Laurdan was added to 1–5 × 10^5^ parasites in LIT medium to the desired final concentration and incubated with gentle rocking in the dark for fifteen minutes. Cells were gently pelleted by centrifugation at 100×*g* for 5 min. The supernatant was carefully removed and the parasites washed in 1×PBS before final resuspension in fresh LIT. The parasites were then transferred to a 4-well chambered coverslip which was then sealed and transferred to the microscope stage.

### 2.3 Bead incubation

To enable imaging in the third channel of the microscope, cells were grown in twelve-well plates on 13 mm diameter coverslips to a density of 8 × 10^4^ and left to grow overnight under the conditions described above, then Laurdan-stained prior to bead incubation as described above. 1.4 × 10^4^ 4 μm uncoated red sulphate-modified latex beads (Invitrogen, UK) were then added onto the cells and the plate was spun in a plate spinner for 1 min at 300×*g*. Coverslips were then placed inverted onto microscope slides and sealed with nail varnish and imaged immediately.

### 2.4 Fluorescence imaging

**Fig. 1:**
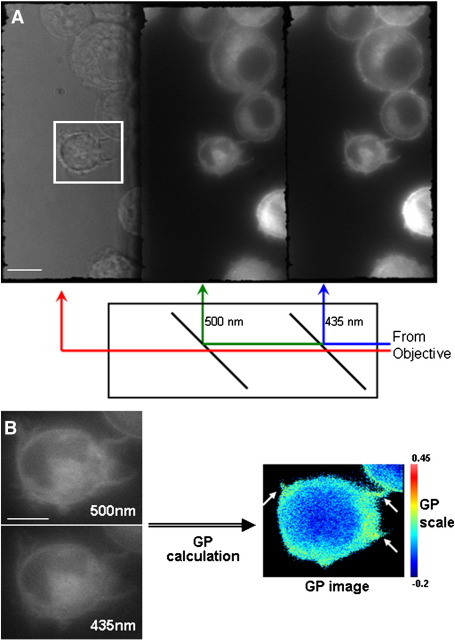
Widefield triplesplit microscope setup. Representation of the Optosplit III block with dichroic mirror set up for Laurdan staining. The two dichroic mirrors separate the fluorescence emission of the Laurdan-stained cells into the blue (435 nm) and green (500 nm) wavelengths, with a third channel showing light from the red end of the spectrum (> 500 nm) (A). Calculation of the generalised polarisation of the cell outlined in (A) showing fluorescence at the blue and green wavelengths (B) and the GP image calculated from these. The range of values represented on the pseudo-coloured GP image is shown as a colourimetric scale to the right of the GP image. Filopodia are highlighted with arrows in the GP image. Scale bars = 10 μm (A) and 5 μm (B).

Samples were excited with a Cairn OptoSource with a 365/10 excitation filter and DM400 dichroic mirror (Cairn Research Ltd, UK) lamp on an Olympus iX71 inverted microscope with a z-axis motorised stage adaptor (Prior Scientific Ltd., UK). An Optosplit III Triple Emission Image Splitter (Cairn Research Ltd, UK) with 435 ± 20 nm and 500 ± 20 nm bandpass emission filters (HQ435/40 and HQ500/40 respectively, Chroma Technology, USA), with 460DCLP and565DCXR dichroic beamsplitters (Cairn Research Ltd, UK) was fitted to the detection port to split the emission image into three wavelengths (see Fig. 1A for a representation of this setup).

Images were captured on an Orca AG split-chip CCD camera (Hamamatsu Photonics, Japan) using Andor iQ software (Andor Technology, UK).

### 2.5 Image analysis

**Fig. 2:**
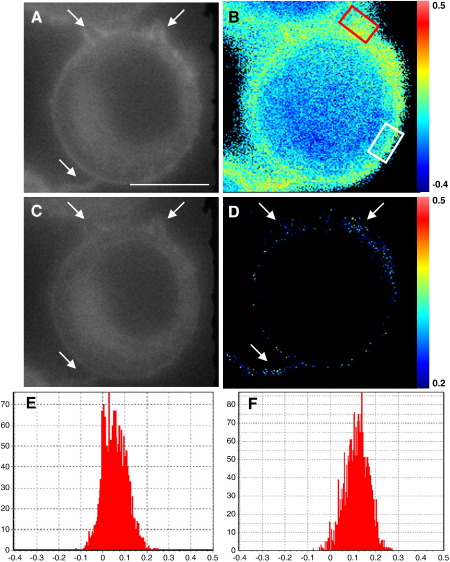
Isolation of discrete high and low generalised polarisation regions within a RAW264.7 cell membrane. Filopodia are highlighted (arrows). Fluorescence of Laurdan stained RAW264.7 cell in the blue (A) and green (C) wavelengths. B) GP of the cell showing the range of the cell's calculated GP values. Regions of high (red box) and low (white box) membrane order are shown. D) GP image showing only those pixels with a GP of 0.2 or more. The regions around the filopodia appear more ordered. GP histograms (x-axis = GP value, y-axis = number of pixels) of the high (E) and low (F) order regions from (B). Scale bar = 5 μm.

Regions of interest (ROIs) of the captured triplesplit images were separated by wavelength channel and aligned using the Cairn Image Splitter plugin for the ImageJ software package [44] and saved as separate files for each wavelength channel. The generalised polarisation (GP) values, images, histograms and GP averages were generated from the blue and green channels using the SimFCS software (Laboratory for Fluorescence Dynamics, CA, USA). Images were not altered before GP calculation. Thresholds were evaluated for each image and set to exclude background fluorescence from the GP calculations and to include only the fluorescence from the cells. Examples of captured images before background subtraction are shown in Figs. 1 and 2. To compensate for any difference in sensitivity between the channels, the G factor was calculated as described by Gaus et al. [45] with 10 μM Laurdan in DMSO, and applied to the GP calculations.

### 2.6 Cholesterol depletion

Cholesterol depletion of cells was performed essentially as described in Gaus et al. [35]. Briefly, cells were incubated for one hour in serum-free DMEM before methyl-β-cyclodextrin (MβCD) (100 mM stock solution in deionised H_2_O) was added to the required concentration, with deionised H_2_O as a control, and incubated for a further one hour. The culture medium was then replaced with fresh serum-free DMEM before staining with Laurdan.

### 2.7 Statistical analysis

GP average of the cell membrane in each GP image was calculated using SimFCS [46]. The GP averages of twenty to forty images were taken for each MβCD treatment and an average and standard deviation calculated per treatment. Statistical analysis was carried out using one-way Analysis of Variance (ANOVA) with Tukey-HSD follow-up tests. Results were considered statistically significant if *p* < 0.05.

## 3 Results and discussion

### 3.1 Optimisation of Laurdan staining

Initial Laurdan-staining experiments were performed using the MDCKII cell line to find optimal concentrations of Laurdan for use with the triplesplit fluorescent widefield microscope setup. Laurdan has been used previously in the range of 2–8 μM for two-photon microscopy [35]. For our new widefield microscope set-up, we started optimising the Laurdan concentration to be used within this range, initially using concentrations of 2–20 μM Laurdan. The criteria for optimisation were for the incorporated Laurdan to fluoresce at sufficient intensity to allow membrane dynamics to be imaged with minimal internal staining by the probe through endocytosis, and an exposure time that minimised photobleaching. Incubation with 10 μM Laurdan for fifteen minutes was the lowest concentration coupled with the shortest incubation period that consistently gave stable measurable fluorescence throughout the plasma membrane with little incidence of internal staining and with an exposure time of under 500 ms which was necessary to reduce photobleaching to a manageable level. When cells incubated with 10 μM Laurdan were optically sectioned (and deconvolved), staining was predominantly at the plasma membrane but this was in contrast to longer (> 20 min) incubations or higher (> 15 μM) Laurdan concentrations where internalisation of Laurdan and subsequent labelling of internal membrane structures were evident. Conversely, concentrations of Laurdan below 10 μM or incubation times below 15 min resulted in insufficient fluorescence or required too long an exposure time. Using the lowest workable concentration also enabled us to minimise the amount of DMSO added with the Laurdan, reducing any physiological effects of this solvent on the cells. Washing the cells after Laurdan staining also helped to prevent internal labelling, removing excess, unincorporated Laurdan and enabled imaging for sixty minutes before internal structures started to show labelling, which was more than sufficient for our experimental purposes.

The setup of the widefield triplesplit microscope with the arrangement of dichroic mirrors is represented in Fig. 1A as used to image a live, Laurdan-stained culture of RAW264.7 cells. In the Optosplit III block, dichroic mirrors separate off first the blue and then the green wavelengths coming from the objective lens. Light of greater than 500 nm wavelength can then be visualised in the third imaging channel, including brightfield imaging with illumination from a lamp passing first through a red filter. To verify whether this new experimental setup can produce useable reproducible ratiometric GP images enabling visualisation of regions of increased or decreased membrane order, we used the RAW264.7 cell line; macrophage-like cells that produce filopodia. These filopodia have been previously shown to possess increased membrane order compared to other regions of the cell membrane [35,39] and so enabled us to verify whether our new system could detect differently ordered regions that have been shown under two-photon microscopy to exist. Fig. 1B shows the image data used in the process of calculating the GP of a RAW264.7 cell. The intensity of each pixel in the blue and green fluorescent channels was used in the GP calculation, with background fluorescence excluded via thresholding. The resultant GP image is shown alongside (Fig. 1B), pseudo-coloured so that pixels with higher GP value pixels appear more red, those of lower GP value more blue, with the background coloured black. The filopodia highlighted with arrows in the GP image can be seen to have a higher GP value, containing more pixels towards the red end of the LUT spectrum. This is similar to the results seen previously with macrophages [35,37] and provides evidence that the new set up with the widefield microscope is sensitive enough to differentiate between regions of varying order.

### 3.2 Discrimination of separate regions within a cell membrane

Differences in the GP of regions within individual cells can also be shown using our setup. The fluorescent images of a live Laurdan-stained RAW264.7 cell show developing filopodia (Fig. 2A and C). When the GP is calculated these appear to have a higher order, or less fluidity, with more yellow and red pixels than other regions of the cell membrane (Fig. 2B). This can be seen more clearly when the low GP-value pixels are excluded, showing only those of a GP of 0.2 or higher (Fig. 2D). Then, the filopodia can be seen to contain more of the pixels at these values than other regions of the cell. Our setup is therefore sensitive enough to show a difference in GP within an individual cell's membrane. We can isolate two equally sized regions that appear in the initial GP calculation to contain regions of greater and lesser membrane fluidity (Fig. 2B, white and blue and red boxes respectively). Histograms of the GP value per pixel of the membrane in each area show the region away from the filopodia displaying apparent lower membrane order (Fig. 2E) with a lower GP average of 0.058 than the region around the filopodia (Fig. 2F) which has a GP average of 0.118, with a shift to the right of pixel values on the histogram.

### 3.3 Cholesterol depletion of Laurdan stained cells

**Fig. 3:**
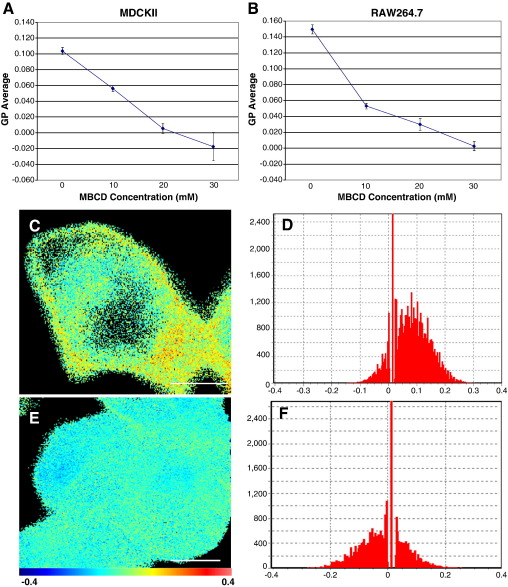
Cholesterol depletion reduces membrane order of MDCKII and RAW264.7 cells. An increase in MβCD resulted in a decrease in average GP in both MDCKII (A) and RAW264.7 (B) cells. *N* for (A) and (B) is at least 20 cells per MβCD concentration. GP image and Histogram of MDCKII cells treated with 0 mM MβCD (C and C respectively) and 20 mM MβCD (E and F). Histograms show GP value (x-axis) and number of pixels (y-axis) at that GP value. Scale bars = 4 μm (C and E).

Previous reports using Laurdan staining with two-photon microscopy have shown a relative lowering of GP average in cholesterol depleted cells [40], where the more ordered regions, including the hypothetical lipid rafts, contain an increased concentration of cholesterol [4]. To test the ability of our system to similarly detect the effects of cholesterol depletion on membrane order we used the cholesterol depletor methyl-β-cyclodextrin (MβCD). Previously, concentrations of 15–35 μM of MβCD have produced a significant reduction in GP average [40] and so we used a similar range of MβCD concentrations (0, 10, 20 and 30 μM). We measured the effect of MβCD on the GP averages of two separate cell lines, macrophage-like RAW264.7 and epithelial MDCKII cells. At least twenty cells per treatment, from three separate experiments, were analysed for both cell lines. The GP average of both the MDCKII and RAW264.7 cell lines decreased with increasing concentrations of MβCD (Fig. 3A and B respectively). Analysis of Variance (ANOVA) with follow-up Tukey-HSD testing showed this reduction in GP average to be significant (*p* < 0.01) in both cell lines. The GP average of MDCKII cells treated with 30 mM MβCD was not significantly lower from those treated with 20 mM MβCD (*p* > 0.05). These GP average curves very closely resemble those previously reported [40] indicating the viability of using our system to study changes in GP in response to added experimental factors.

An example of the effect on GP of MβCD is shown in Fig. 3. An MDCKII cell with just the water vehicle added (0 mM MβCD) shows the GP range over its membrane as seen in untreated cells (Fig. 3C) having a GP average of 0.082, with the distribution of GP values for each pixel shown in Fig. 3D. With a cell that has been incubated with 30 mM MβCD, the resultant GP displays a lower order across the membrane (Fig. 3E) with a GP average of − 0.017 and fewer areas showing lateral organisation. The reduction in the GP distribution toward a more negative mean value in the MβCD-treated cell compared to the control can be seen in the histograms of GP value per pixel (comparing Fig. 3D and F).

### 3.4 Visualisation of cellular structures

**Fig. 4:**
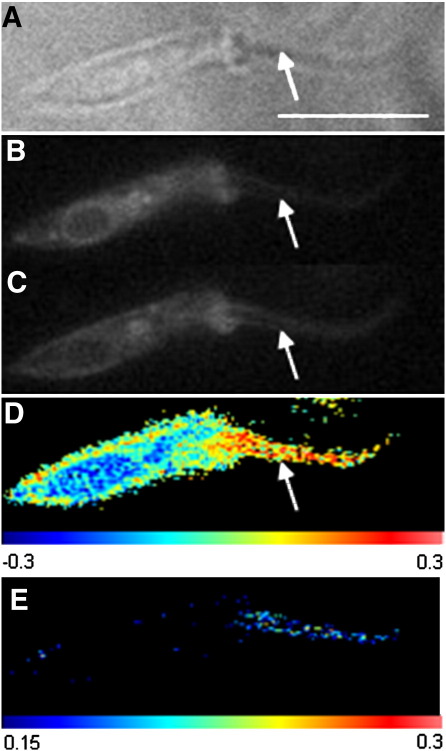
Laurdan staining of *Trypanosoma rangeli*. Far-red illuminated brightfield (A) and fluorescent images (B and C) of a Laurdan-stained *T. rangeli* using the triplesplit widefield microscope. GP analysis (D) shows the more highly ordered flagellum (arrow) when compared to the rest of the cellular membrane. Exclusion of low GP pixels (E) demonstrates the greater number of high-GP pixels in the flagellum. Scale bar = 4 μm.

Structures such as functional cell membrane protrusions have previously been shown to contain membranes of increased order, such as the demonstration that the primary cilia in epithelial cells possess a l_o_ domain at their base [41]. Flagellar membranes have also been shown by Tyler et al. [42] to be more highly ordered regions than other regions of the cellular membrane, and this can be seen for the American trypanosome *Trypanosoma rangeli*, a flagellated protozoon (Fig. 4). The red-filtered brightfield image of an individual Laurdan-stained Trypanosome (Fig. 4A) and the 500 nm (Fig. 4B) and 435 nm (Fig. 4C) fluorescent images were captured on the triplesplit widefield microscope for analysis. The GP image then generated (Fig. 4D) shows the flagellum to have greater membrane order than the rest of the cell's membrane, as has been previously demonstrated for the African Trypanosome *Trypanosoma brucei* [42]. When regions of low membrane order are excluded, the higher order of the flagellar membrane can clearly be seen (Fig. 4E).

### 3.5 Laurdan photoselection effect in cellular imaging

Although our GP results can be seen to closely match those reported from two-photon microscopy, indicating its potential for live cell imaging with Laurdan in widefield microscopy, there remains the underlying problem of the Laurdan photoselection effect. This effect is intrinsic to this method and arises because Laurdan aligns in the phospholipid bilayer parallel to the acyl chains and with polarized light, excitation is strongest in the plane parallel to the excitation [39]. For confocal microscopy and especially 2-photon microscopy, which utilizes polarized laser excitation, this effect can be strong. For instance, for a homogenous spherical lipid vesicle, a ring of bright fluorescence can be observed with top and bottom being relatively less fluorescent [39]. Compounding this effect are the observations that differences in the phase of the membrane can affect both the magnitude of the effect and that polarized light, which photoselects well-oriented Laurdan molecules, also selects Laurdan molecules associated with high GP values[37]. Thus the photoselection effect can result in the skewing of the calculated values of pixels in the GP image and therefore the GP histogram.

For living cells the potential significance of the photoselection effect in interpretation of GP data, given the large size and irregular geometry of cells and especially when considering phenomena using 3 or 4-dimensional microscopy deserves careful consideration. Parts of the cells will inevitably lie in different orientations relative to the excitation light and this has the potential to cause systematic errors in interpretation which have not, to date, been fully considered. In this paper, we describe the use of Laurdan microscopy on living cells excited by epifluorescence with a non-coherent UV source. There is little existing data with which to gauge the magnitude of the photoselection effect under such conditions, although it has been suggested that non-polarized excitation may give rise to smaller, less predictable effects than two-photon microscopy [37]. Targeted experiments specifically addressing this issue are highly desirable and will be important in gaining increased accuracy from GP calculations associated with live cell imaging.

### 3.6 Utilisation of third channel imaging

**Fig. 5:**
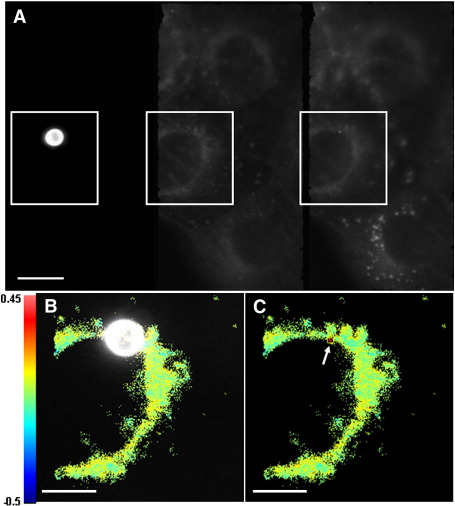
The effect of a BSA-coated bead on membrane dynamics can be visualised using the third channel. Optosplit III image showing all three channels (A). GP analysis of the area outlined in (A) shows the position of the BSA-coated bead against the cell membrane (B) with a discrete region of increased GP seen at the bead's location (C), as indicated by the arrow. GP scale of (B) and (C) shown to the left. Scale bars = 10 μm (A) and 5 μm (B).

Addition of BSA coated 4 μm red beads was seen to increase the order of the cell membrane at the contact points (Fig. 5) analogous to synapses between cells and pathogens. As well as demonstrating the potential of coat proteins to influence the composition of adjacent plasma membrane with which they are in contact, this shows the potential of our system for analysing GP changes in live cells in response to factors or relative to markers that can be followed in the third channel, with images in all three channels captured simultaneously.

## 4 Conclusions

These results demonstrate the utility of a low-cost widefield microscope to effectively replicate results gained from Laurdan staining and two-photon microscopy, in both the heterogeneity of individual cells and protozoan membranes, and in changes in membrane fluidity in response to chemicals that remove the cholesterol from membranes and prevent the cell forming the more ordered regions. The potential is there for this setup to image live cells and monitor the changes in membrane order in response to different factors. In addition to previously reported set-ups, we have also demonstrated the ability of our system to investigate the localisation of red-tagged markers and proteins in relation to high or low GP regions, which can be added effectively onto an inverted microscope, and at less expense compared to the two-photon system.
